# Evaluation of Variability in Dietary Quality of School Lunches Meeting National School Lunch Program Guidelines by Socioeconomic Status and Rurality

**DOI:** 10.3390/ijerph17218012

**Published:** 2020-10-30

**Authors:** Jillian M. Joyce, Richard R. Rosenkranz, Sara K. Rosenkranz

**Affiliations:** 1 Department of Nutritional Sciences, Oklahoma State University, Stillwater, OK 74078, USA; 2 Department of Food, Nutrition, Dietetics and Health, Physical Activity and Nutrition Clinical Research Consortium, Kansas State University, Manhattan, KS 66506, USA; ricardo@ksu.edu (R.R.R.); sararose@ksu.edu (S.K.R.)

**Keywords:** school lunch, dietary quality, child nutrition, socioeconomic status, rurality

## Abstract

Research suggests that the dietary quality (DQ) of school lunches meeting the National School Lunch Program (NSLP) requirements may vary significantly. Possible drivers of variation include factors, such as socioeconomic status (SES) and rurality. The purpose of this cross-sectional study was to determine whether there was variation in nutrient content and DQ by SES and rurality, when analyzing middle school lunch menus meeting NSLP requirements. A random sample of 45 Kansas middle school lunch menus each were obtained from websites of randomly selected districts from low- and high-SES strata. Thirty-day menus were analyzed for nutrient content. Healthy Eating Index (HEI) 2015 scores were calculated for DQ. Rurality was determined for schools by National Center for Education Statistics (NCES) locale. There were significant differences in added sugar (*p* < 0.001) and calcium (*p* = 0.001) favoring high-SES menus, and in sodium (*p* = 0.001) favoring low-SES menus. There were no nutrient differences by rurality. The HEI scores were not different by SES or rurality, with a mean score (SD) 61.9 (2.6) across all schools. Middle school lunch DQ in Kansas does not vary by SES or rurality. Efforts to improve DQ should focus on all foodservice operations, not specifically low-SES or rural schools.

## 1. Introduction

According to the USDA Food and Nutrition Service (FNS), there is agreement among public health practitioners that food insecurity and poor nutrition are major issues, with a need to treat these problems through providing adequate and nutritious food to underprivileged populations [1]. Federal food assistance programs are part of their solution [1]. The National School Lunch Program (NSLP), especially the free and reduced-price lunch benefit, is one such federal food assistance program that seeks to decrease disparities in nutrition among children by providing nutritionally balanced meals at a low cost, or free, available every school day [2,3]. Despite the NSLP’s goal of treating nutrition disparities, recent studies from our lab group found that there is the possibility for significant variation in nutrient content and dietary quality (DQ) of school lunches, while meeting NSLP nutrition standards [4,5]. One previous cross-sectional study compared six weeks of a typical school lunch menu, obtained from an actual school district that was meeting baseline NSLP nutrition standards, with six weeks of a best practice school lunch menu, which was created by a Registered Dietitian with the goal of optimizing nutrition, regardless of feasibility and greatly exceeding baseline NSLP nutrition standards. The results from the study revealed several large, statistically and clinically significant differences in nutrient content and DQ [4]. A second more recent cross-sectional study compared six weeks of elementary school lunches meeting various NSLP nutrition standards as policy has changed, including the School Meal Initiative, Healthy, Hunger-free Kids Act, and Child Nutrition Program Flexibilities, as well as with best practices implemented. The results again indicated the possibility for large, statistically and clinically significant differences in nutrient content and DQ based on policy standards [5]. Dietary quality was highest in both studies with best practices implemented [4,5]. These results presented more questions—does this variation actually exist outside of this theoretical comparison and, if so, what are the possible drivers of this variation?

There has been some research regarding perceived barriers to improving DQ of school lunches, which could provide insight into potential sources of variation. Studies by Nollen et al., (2007), Brouse et al. (2009), and Fulkerson et al., (2002) investigated perceived barriers to improving DQ of school lunches, and found two common themes, including that (1) schools are doing the best they can with available resources and (2) that there are financial pressures and concerns [6,7,8]. With these themes in mind, the socioeconomic status (SES) of school districts presents as a possible driver of variation in nutrition provided by school lunches, if variation does exist.

Elsewhere in public health, SES, or income level and wealth, has been shown to be a source of disparity in child and adolescent nutrition. A narrative review by Hanson et al. (2007) was performed to determine associations between SES and five health behaviors during adolescence, including diet and nutrition [9]. Twenty-five of the 31 articles that were included in this review indicated that there were associations between low SES and inadequate fruit and vegetable intake, as well as higher fat and refined grain intake in adolescence [9]. The overall evidence indicated a disparity in general adolescent diet by SES. When specifically considering school-aged children, a cross-sectional study conducted by Fahlman et al. (2010) investigated differences in the overall diet of 7–12th graders from low-SES, urban and high-SES, suburban Michigan schools in a health education class [10]. The results showed that lower SES students were more likely to consume higher amounts of meat, fried foods, and empty calories; were less likely to consume fruits and vegetables, dairy products, and grains; had lower self-efficacy to make healthy diet choices or changes; and, had less overall diet knowledge than their higher SES counterparts [10]. These differences showed a large disparity in dietary behaviors, knowledge, and self-efficacy of schoolchildren by SES. Narrowing in on the school food environment, Delva et al., (2007) performed a large cross-sectional study with a nationally representative sample of American schools, investigating ethnic and SES differences in the availability of healthful food choices [10]. Parent education was the proxy measure that was utilized to determine student SES. The results revealed a negative linear relationship between SES and schools offering breakfast, and the percentage of students participating in NSLP and Team Nutrition programs. There was a non-significant, positive trend for an association between SES and number of more-healthful foods available. Lower SES schools also had a significantly higher number of days with fast-food items for lunch, a lower number of more-healthful food items available a la carte, and a lower ratio of more-to-less healthy foods available to students (i.e., a less-healthful mix of available options) [11]. These results showed a variation in DQ of the overall school food environment by SES. Together, these studies suggest that SES may be an important driver of DQ variation in child and adolescent overall diet, schoolchildren’s overall diets, and overall school food environment. However, no known studies have investigated the potential variation in nutrition that was provided by NSLP-qualifying lunches, which have broad reach in adolescence and where efforts to improve school lunch DQ may have the greatest impact.

Related to SES, rurality has also been shown to play a significant role in many health disparities, including nutrition-related issues. A cross-sectional study conducted by Davis et al., (2011) evaluated overweight, obesity, and related health behaviors in rural and urban children while using NHANES data. The results of the study indicated that urban and rural areas were significantly different in most demographics, including SES, with urban residents being of higher SES than rural residents [12]. There were no differences in the dietary intake between urban and rural participants in this study; however, there was a significant difference in obesity prevalence, with rural children significantly more likely to be obese than urban children, 22% vs. 17%, respectively. There were also significant differences in predictors of obesity between urban and rural participants. Rural obesity was predicted by race, physical activity level, and screen time, while urban obesity was predicted by race, age, SES, and dietary intake [12]. Another study supporting rurality as a possible driver was a cross-sectional study conducted by Befort et al., (2012), investigating differences in obesity and behavioral determinants of obesity of adults by residence using NHANES data [13]. This study found that rural residents reported lower income levels, had significantly higher consumption of calories from fat, and had significantly higher obesity prevalence than urban residents. Socioeconomic status modified the strength of these relationships found between rural and urban residents [13]. Additional studies showed associations between rurality and risk factors for disease, disease prevalence, and mortality [14,15,16]. These studies show the importance of investigating potential differences between low and high SES, and rural and urban school districts with regard to the DQ of school lunches in order to determine the potential disparities that may indicate a need for intervention.

With the questions presented above, the purpose of the current study was to determine whether there are differences in nutrient content and DQ provided in middle school lunches, across the state of Kansas, in high versus low-SES and in rural versus urban school districts. We hypothesized that there would be significant differences in nutrient content and DQ of middle school lunch menus, favoring higher SES, less rural school districts.

## 2. Materials and Methods

The current study was a cross-sectional content analysis, comparing lunch menus from a large sample of randomly selected middle schools in Kansas by SES and rurality.

### 2.1. Socioeconomic Status

The socioeconomic status of school districts was determined while using the percentage of schoolchildren in the district receiving free or reduced-price lunches (FRPL). The researchers obtained a list of all school districts in Kansas and the percentage of the students receiving FRPL from the Kansas Department of Education K–12 Report Generator [17]. Data were grouped by district/organization totals for all schools during the school year of 2015–2016, and then used for district SES stratification and assignment. The districts were ranked from lowest to highest percent FRPL. Given the best fit for the data, districts with >50% FRPL were assigned to the low-SES strata and districts with <50% FRPL were assigned to the high-SES strata. The low-SES strata contained 153 districts (53.5% of total), and the high-SES strata contained 130 districts (45.6% of total). Three districts had 50.0% FRPL and they were excluded from analyses. Research assistants were blinded to SES and school district stratification in order to reduce potential bias.

### 2.2. Rurality

Rurality was determined while using the locale reported for each school district in the US by the National Center for Education Statistics (NCES) [18,19]. The school district name from the Report Generator was entered into the NCES “Search for Public School Districts” search engine [18]. Once the school district was found using the search engine, the locale was obtained from the district’s directory profile. Locales include city, suburb, town, and rural, and they were developed by NCES based on proximity to metropolitan areas, population size, and population density [19]. The NCES created locale codes for research and data reporting related to schools. These codes have not been validated, but they do use similar base information in order to determine designations as other coding systems [19]. Locales were mutually exclusive and coded 0 for city, 1 for suburb, 2 for town, and 3 for rural.

### 2.3. Sample

Once the strata were created, including all Kansas school districts, school district USD identification numbers were randomized for each stratum. The first 68 randomized school districts were selected, with a goal of 90 total school districts with complete and usable data for analysis in order to obtain a representative sample. Menus were obtained for school districts’ middle schools from their publicly available websites. Publicly available menus were used to obtain food item information, as it would not be feasible to obtain detailed production records and purchased product information from all school districts in such a large sample. All of the menus were obtained for the first six weeks of the 2016–2017 school year, to control for variations in seasonality of menus. School districts were excluded if they did not have menu information available on their website and if the information on the menu obtained was not complete enough for analysis (i.e., food items listed too generally, only one week available, unable to extract information from the website, etc.). School districts were also excluded if the publicly available menu was not current.

### 2.4. Nutrient Content

**Figure 1:**
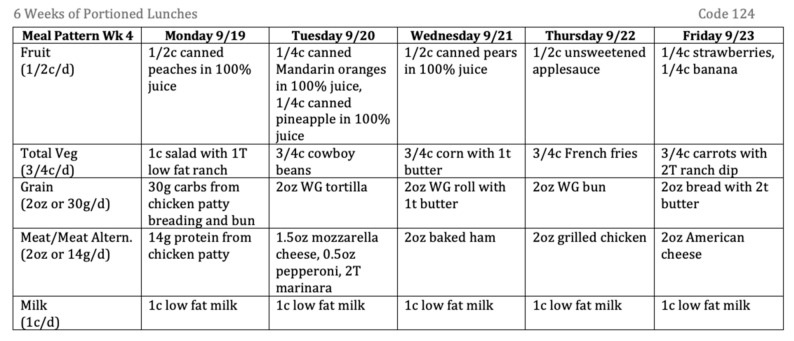
Image of a one-week sample of a portioned middle school lunch menu.

The first six weeks (30 days) of each school district menu were portioned per NSLP nutrition standards for the middle school age group [20]. Figure 1 illustrates an example of a week of portioned menus. Because there was not access to specific product information, a system of assumptions about food items was created. Assumptions about foods served were made based on common types of foods and other information available on menus and in favor of the school districts, such that there would be more favorable nutrient content and higher DQ following analysis. Supplement S1 depicts a comprehensive list of assumptions made during portioning of menus. Multiple research assistants completed menu portioning. To maximize inter-rater reliability, the principal investigator that trained all researchers was present during all portioning work time, maintained a list of assumptions on-hand for reference, and reviewed all completed portioned menus.

Once all of the menus were portioned per NSLP middle school age group nutrition standards, the portioned menus were entered into ESHA Food Processor Nutrient Analysis Software (ESHA Research, version 4.1.1255, Salem, OR) in order to determine nutrient content of all major macro- and micro-nutrients. Because specific food item information was not available, assumptions also had to be made during nutrient analysis, based on the expert opinion of the principal investigator, a Registered Dietitian with experience in childcare menu development, regarding foods typically and realistically served in schools, and such that school districts had more favorable nutrient content and DQ. Because foods can be searched for in ESHA while using ESHA codes, one code was selected for each common food item used in nutrient analysis. These codes were then used to input common food items into the Food Processor. This maximized inter-rater reliability and minimized variation due to different forms of the same food item being analyzed (i.e., one ESHA code for steamed broccoli as opposed to several different forms of steamed broccoli being used). A list of ESHA codes used can be found in Table S2. In order to further increase inter-rater reliability, the principal investigator again trained all researchers on nutrient analysis methods, checked data input during training and periodically throughout analysis, was present during all analysis sessions, and spot-checked nutrient analysis during DQ and further data analysis.

### 2.5. Dietary Quality

Dietary quality was calculated following menu portioning and nutrient analysis while using the HEI 2015 [21]. An Excel calculator was created in order to calculate HEI 2015 scores. A list of HEI calculation equations and instructions for DQ analysis used in the current study can be found in Supplement S2. The HEI is a valid and reliable measure of DQ that measures compliance with Dietary Guidelines for Americans recommendations for a healthy diet [22].

### 2.6. Statistical Analysis

Statistical analysis was completed while using SPSS Statistical Software (IBM Analytics, version 23, Armonk, NY, USA). Descriptive statistics were determined for SES and rurality groups, including averages and standard deviations of nutrient content and HEI score, and parametric assumptions were checked. Independent *t*-test and two-way ANOVA were used in order to determine the main and interaction effects of SES and rurality on nutrient content and DQ. Chi-squared was used to determine differences in characteristics of menus, including distribution of SES and rurality groups. Effect size was calculated while using Cohen’s *d* and partial eta squared. Bonferroni correction was used for multiple comparisons.

## 3. Results

**Figure 2:**
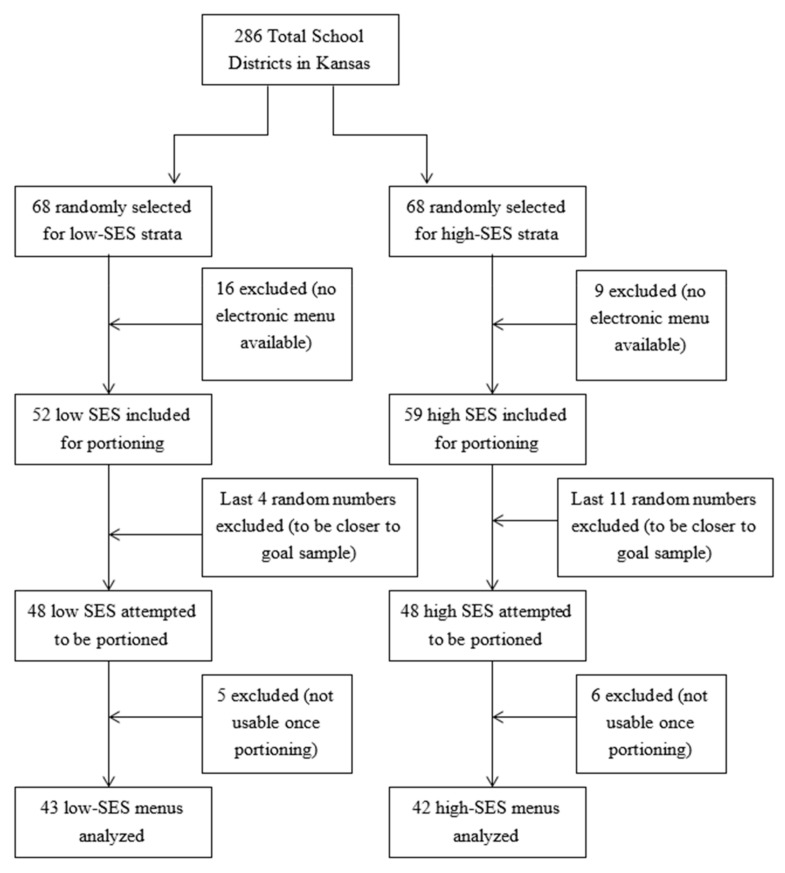
Flow chart of final sample selection and inclusion.

Initially, 68 school districts were randomly selected from the low- and the high-SES strata, 136 districts in total, with the goal of including 45 menus from each stratum in the final analysis. Of these 136 total initial school districts sampled, 25 school districts’ publicly available menus did not have adequate detail for analyses, 16 low SES, and nine high SES. Thus, 111 school districts produced menus that appeared to be initially usable from their publicly available websites, 52 low SES, and 59 high SES. With the goal of 90 menus, the last additional random numbers on each strata’s list, four low-SES and 11 high-SES menus, were not included, leaving 48 menus from each stratum for portioning with three menus per strata remaining for oversampling. Once portioning began, due to lack of specific or usable information, five low-SES and six high-SES menus were not able to be portioned and, thus, analyzed, resulting in a total of 85 menus portioned (43 low-SES and 42 high-SES). With 30 days of lunches analyzed per menu, this analysis included 2550 lunches. Figure 2 depicts a flow chart of final sample selection and inclusion.

**Figure 3:**
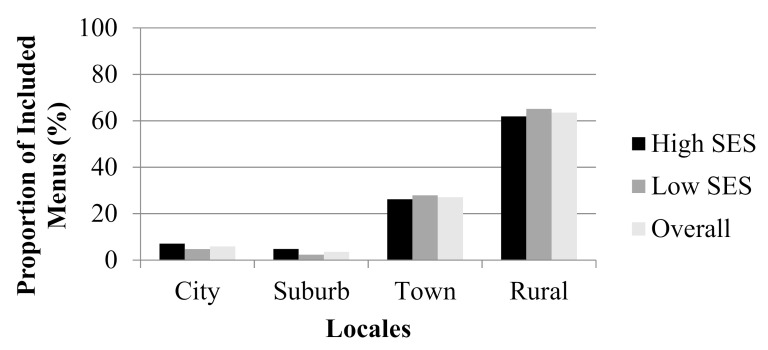
Proportion of included menus by locale. There were no significant differences between strata or overall in proportion of menus by locale (*p* > 0.05).

Of the 85 menus included in analyses, 50.6% were low SES and 49.4% were high SES. The high-SES strata had a mean (±SD) percent FRPL of 32.3 ± 10.2% (range: 8.3–48.8%). The low-SES strata had mean (±SD) percent FRPL of 58.4 ± 6.8% (range: 50.3–78.7%). The proportions of menus in each stratum and overall by locale can be found in Figure 3. There were no significant differences in the proportions of school district SES or in proportions of rurality between all, low-SES, or high-SES menus.

Table 1 depicts low and high SES overall means and standards deviations for nutrient content and DQ. There were several small to moderate, significant differences by SES. Menus were significantly different in nutrient content by SES, including added sugar (difference (high–low) = −0.4 g or −80%, *d* = 0.777, *p* < 0.001), calcium (difference (high–low) = 5.3 mg or 1%, *d* = −0.223, *p* = 0.001), and sodium (difference (high–low) = 54.1 mg or 48%, *d* = −0.657 *p* = 0.001). Differences were such that the high-SES menus had lower added sugar, higher calcium, and higher sodium content. There was no significant difference in HEI score, or DQ, between low- and high-SES menus.

Table 2 illustrates the overall means and standard deviations for nutrient content and DQ by rurality. There were no significant differences in the nutrient content or HEI scores for DQ by rurality.

There was a significant interaction effect between rurality and SES for nutrient content, but not for HEI score. A significant interaction effect was seen for calcium (*p* = 0.001). This interaction was such that the difference in calcium favoring high-SES menus diminished as the menu became more rural (difference (high SES–low SES): city = 69 mg, suburban = 41 mg, town = 19 mg, and rural = −7 mg) and reversed for the rural locale menus, such that the low-SES menus had higher calcium content than the high-SES menus by 7 mg.

In addition to statistical analysis, several general/overall observations were made while calculating HEI scores for DQ. HEI scoring components consist of total fruit, whole fruit, total vegetable, dark green vegetable and legumes, whole grains, dairy, total protein foods, seafood and plant proteins, fat ratio, refined grains, sodium, added sugar, and saturated fat. Most of the menus received a maximum score for total fruit and total vegetable (overall mean HEI component score ± standard deviation: total fruit 4.8 ± 0.1 out of 5, total vegetable 4.9 ± 0.1 out of 5) in meeting NSLP nutrition requirements, unless the menu greatly exceeded NSLP allowable calorie amounts, as HEI scores are standardized to calorie amounts. The majority of menus received a score of zero, or mostly scores of zero, for the whole fruit component (overall mean HEI component score ± standard deviation: 2.1 ± 1.4 out of 5), as canned fruit tended to be the fruit option of choice. Most of the menus received the maximum score for dark greens and legumes on two days per week, as dark green vegetables and legumes are two of five required varieties of the vegetable meal component that must be provided over the course of the week (overall mean HEI component score ± standard deviation: 1.9 ± 0.3 out of 5). Most of the menus received a score of zero for the whole grain component (overall mean HEI component score ± standard deviation: 2.1 ± 1.9 out of 10), as most menus provide whole grain-rich grains and not whole grains. The exception to this observation was that many menus included corn grain products, which were often whole grain (i.e., corn chips, hard taco shells, cornbread, corndogs). Most of the menus received the maximum score for dairy and total protein foods in meeting the NSLP nutrition requirements (overall mean HEI component score ± standard deviation: dairy 10.0 ± 0.1 out of 10, total protein foods 5.0 ± 0.0 out of 5), unless the menu greatly exceeded NSLP allowable calorie amounts, as the HEI scores are standardized to calorie amounts. The majority of menus received a score of zero for the seafood and plant protein component (overall mean HEI component score ± standard deviation: 0.1 ± 0.2 out of 5), as few menus included these items as a meat/meat alternate food item. If seafood or plant proteins were included, they generally consisted of bean burrito, fish sticks or fish patty sandwich, peanut butter, hummus, or tuna salad. With regard to the fatty acid ratio, saturated fat, and sodium components, most menus received a wide range of scores, generally on the lower/less favorable end of the range (overall mean HEI component score ± standard deviation: fatty acid ratio 2.0 ± 0.6 out of 10, saturated fat 5.1 ± 0.6 out of 10, sodium 3.9 ± 0.8 out of 10). Because of NSLP nutrition standards at the time of the analysis and the assumptions made, all menus received the maximum score for the refined grain component and for the added sugar component (overall mean HEI component score ± standard deviation: refined grain 10.0 ± 0.0 out of 10, added sugar 10.0 ± 0.0 out of 10).

## 4. Discussion

This cross-sectional study included an analysis of the nutrient content and DQ of 85 randomly selected school districts’ middle school lunch menus, or 2550 school lunches, in Kansas. The menus were compared in order to determine whether there were differences in DQ provided in middle school lunches in high versus low SES and in rural versus urban school districts. Across all schools, the overall mean HEI score was 62, which—according to the USDA CNPP— “needs improvement” [23]. The results showed that there were no significant differences by SES or by rurality in DQ. The results also showed that there were few main effects or interaction effects on nutrient content by SES and rurality. Menus differed in added sugar, calcium, and sodium by SES. The differences in added sugar and calcium favored the high-SES menus, while the difference in sodium favored the low-SES menus. Despite minimal significant differences between menus, the differences in added sugar and sodium were nearly large, at 0.777 and −0.657, respectively, based on effect size. Menus did not differ by rurality alone. However, there was one difference due to the interaction of SES and rurality, in calcium content, such that, as the school district became more rural, the difference in calcium content diminished to the point that, in the most rural districts, lower SES menu calcium content exceeded higher SES menu calcium content. Overall, it does not appear that middle school lunch menus in Kansas differ significantly in the nutrient content or DQ by SES or rurality. However, there is room for improvement in DQ across SES and rurality overall.

Several previous studies have indicated that there are significant differences in dietary behavior, dietary knowledge, and self-efficacy to consume a healthy diet, in schoolchildren and also in the overall school food environment, by SES [10,11]. However, the current study differs significantly from these other studies, in that the current study focuses on the reimbursable meal, not overall schoolchild diet or overall school food environment. This is likely the reason for the difference in results, as the reimbursable lunch investigated here is well regulated, while overall child diets are not regulated (directly) and competitive school foods are much less regulated. This is the first known study to investigate associations between school nutrition and rurality. There have been other, more general population studies that have found significant differences in nutrition, disease prevalence, weight status, and other health behaviors by locale [12,13,14,15,16]. With these studies indicating the possibility for variation in nutrition by rurality [12,13,14,15,16], in conjunction with previous research by our lab group indicating the possibility for significant variation in DQ of school lunches meeting NSLP nutrition standards [4], it was important to investigate the differences in school nutrition that are associated with rurality, especially as federal food assistance programs, including the NSLP, seek to eliminate disparities in nutrition [1]. Again, the lack of significant differences in DQ by rurality in the current study is likely due to the fact that the NSLP regulates the nutrition that is provided by participating schools’ lunches. Previous research indicates that the 2012 NSLP guidelines provide a DQ score of about 75 as a baseline just for meeting the requirements [4]. The DQ score provided by meals analyzed in the present study was 62, which is lower than 75 provided by meeting baseline NSLP requirements. This difference could be due to schools not meeting NSLP requirements, or potentially due to assumptions that were made by the researchers. Further, more in-depth investigations would be needed for each individual menu to clarify the reason for the lower DQ score.

There were several strengths to the current study. First, there was a large sample size, 85 total menus and 2550 school lunches, randomly selected from Kansas school districts. There were numerous quality control measures taken in order to eliminate sources of bias and reduce the error due to researchers and methodology. For example, assumptions were made in favor of better nutrition in schools’ lunches and, thus, significant differences were less likely to be found and, if found, were more likely to be due to the foods served, and not due to error in assumptions. The principal investigator trained and monitored all researchers on all aspects of data analysis to increase inter-rater reliability. Lists of assumptions for portioning and of ESHA food codes increased inter-rater reliability, favored higher DQ in school lunches, and provided methodological consistency and transparency. Checking all portion records and spot-checking of nutrient analysis while completing, during data formatting in Excel, and during HEI calculations also increased inter-rater reliability.

There are also several limitations to the current study. Despite having a large sample size for nutrient content comparisons, post-hoc power analysis indicates that power may not have been adequate to detect differences in DQ between SES strata or locales. Numerous assumptions had to be made throughout data analysis due to lack of specific school food item information. It was not realistic to obtain this information for the sample size included. This limitation was minimized by consistent and documented assumptions; however, giving the schools the benefit of the doubt may have also masked any true differences or disparities by SES or rurality that do exist. Another limitation was that there were multiple researchers performing data analysis. Again, numerous control measures were taken in order to ensure optimal consistency in analysis by researchers. An additional limitation was the use of percentage of students receiving FRPL as a proxy for SES of school districts. However, according to the NCES, the percentage of FRPL is reported to be the best and most commonly used proxy [24]. There is a strong correlation between the percentage of FRPL and school district SES, as they are both determined by family income level. The percentage of FRPL provides information on relative SES [24]. According to a report by Cruise and Powers (2006), looking at the relation between FRPL eligibility counts by the NCES and poverty estimates by the 2000 Census, the percentage of FRPL may be the most current, reliable, and direct measure of sub-county, low-income status for children and school districts, as FRPL provides information on an even smaller area than the Census, which does not look smaller than the county level [25]. Additionally, according to a cross-sectional study examining the associations between the percent of students receiving FRPL and other community-based SES measures, percent FRPL was significantly, strongly, and consistently associated with percent of families in poverty, percent of households in poverty, and median household income [26]. Thus, the percentage of students receiving FRPL was used to measure SES of school districts in this study. Finally, there was relatively small separation between the high and low-SES strata in terms of the percentage of students receiving FRPL. This was unavoidable due to the nature of the FRPL distribution for the state of Kansas, and in order to obtain an adequate sample size for comparison.

The DQ observations made throughout menu analysis by the principal investigator provide valuable information moving forward. Scoring components that could use improvement include increased whole fruit (five points), increased whole grains (10 points), increased seafood and plant proteins (10 points), decreased added sugar (10 points), decreased sodium (10 points), decreased saturated fat (10 points), increase in healthy to unhealthy fat ratio (10 points), and ensuring that calories remain within NSLP nutrition standards. Changes to one or two of these scoring components could raise the average HEI score by five, 10, 15, or even 20 points. Based on the overall average HEI score of approximately 62 for this study, changes to two or three of the HEI scoring components in need of improvement could raise HEI scores to or above 80 points and be considered “good”, while also setting the national standard. Previous research by our lab indicates that, if the best practices are also implemented in menu planning, the HEI scores could be as high as 90–95/100, which is very good [4,5].

Future research is needed in several areas. There is limited research investigating the DQ of school lunches and the overall school food environment. Additionally, studies that do exist evaluate DQ cross-sectionally. There is a need for longitudinal studies with more detailed food product information in order to track trends and impact of policy changes. There is also limited research on how to improve the DQ of school lunches. As mentioned above, there are some areas where improvements can be made with small changes to current menus.

## 5. Conclusions

Overall, there do not appear to be meaningful differences in the nutrient content or DQ when analyzing a large sample of Kansas middle school lunch menus by SES or rurality. These are positive results, as this indicates that the NSLP as a public health nutrition program to eliminate disparities appears to be working, and it appears that children of all SES and regional locales in Kansas are likely receiving similar nutrition via school lunches. These results also suggest that initiatives to improve school lunch DQ should focus on all schools equally, but may be particularly important in areas where opportunities for high DQ outside of the school food environment may be limited. Finally, the results suggest that, if widespread improvements in DQ of school lunches were desired, it would be appropriate to focus on improving DQ through NSLP policy changes.

## Figures and Tables

**Table 1:** Comparison of nutrient content and dietary quality (DQ) by socioeconomic status (SES).

Nutrient	Low SES (Mean ± SD)	High SES (Mean ± SD)	*p*-Value	Cohen’s *d*
Calories	611 ± 22	615 ± 22	0.304	−0.182
Protein (g)	30.4 ± 0.8	30.5 ± 0.8	0.245	−0.125
Carbohydrate (g)	74.9 ± 4.0	74.8 ± 3.3	0.189	0.027
Total Fiber (g)	7.7 ± 0.5	7.6 ± 0.5	0.853	0.200
Sugar (g)	32.1 ± 2.2	31.7 ± 2.1	0.198	0.186
Added Sugar (g)	0.5 ± 0.7	0.1 ± 0.2	0.000 *	0.777
Total Fat (g)	22.1 ± 1.3	22.4 ± 1.4	0.898	−0.222
Saturated Fat (g)	8.1 ± 0.4	8.2 ± 0.6	0.781	−0.196
MUFA (g)	5.7 ± 0.6	5.7 ± 0.6	0.819	0
PUFA (g)	3.4 ± 0.5	3.4 ± 0.4	0.638	0
Trans Fat (g)	0.6 ± 0.1	0.6 ± 0.2	0.110	0
Cholesterol (mg)	66.5 ± 5.7	67.6 ± 6.2	0.709	−0.185
Vitamin A (IU)	3480.9 ± 980.5	3314.0 ± 1088.8	0.115	0.161
Thiamin (mg)	0.38 ± 0.03	0.38 ± 0.03	0.822	0
Riboflavin (mg)	0.74 ± 0.04	0.73 ± 0.03	0.613	0.283
Niacin (mg)	5.44 ± 0.58	5.47 ± 0.60	0.520	−0.051
Vitamin B6 (mg)	0.51 ± 0.05	0.51 ± 0.04	0.912	0
Vitamin B12 (mcg)	1.87 ± 0.12	1.88 ± 0.11	0.255	−0.087
Biotin (mcg)	1.70 ± 0.60	1.89 ± 0.78	0.036	−0.273
Pantothenic Acid (mg)	1.78 ± 0.10	1.80 ± 0.11	0.124	−0.190
Folate (mcg)	77.56 ± 9.15	76.02 ± 7.88	0.144	0.180
Vitamin C (mg)	26.75 ± 6.04	27.88 ± 6.31	0.391	−0.183
Vitamin D (IU)	8.11 ± 2.75	9.93 ± 4.18	0.028	−0.514
Vitamin E (mg)	1.51 ± 0.19	1.52 ± 0.25	0.610	−0.045
Vitamin K (mcg)	29.94 ± 7.34	29.95 ± 9.10	0.656	−0.001
Calcium (mg)	494.9 ± 22.0	500.2 ± 25.5	0.001 *	−0.223
Fluoride (mg)	0.02 ± 0.00	0.02 ± 0.00	0.060	0
Iron (mg)	3.58 ± 0.24	3.56 ± 0.22	0.992	0.087
Magnesium (mg)	92.88 ± 5.62	92.62 ± 4.85	0.440	0.050
Phosphorus (mg)	515.72 ± 24.40	518.59 ± 23.85	0.381	−0.119
Potassium (mg)	1019.0 ± 63.6	1023.5 ± 38.5	0.291	−0.086
Sodium (mg)	1064.9 ± 82.5	1119.0 ± 82.2	0.001 *	−0.657
Zinc (mg)	3.69 ± 0.31	3.73 ± 0.25	0.523	−0.142
HEI ^^^	62.4 ± 2.5	61.6 ± 2.7	0.097	0.307

* Results were significant for *p* < 0.001. ^^^ HEI score out of 100 points maximum, with a higher score indicating higher DQ.

**Table 2:** Comparison of nutrient content and DQ by rurality.

Nutrient	City (Mean ± SD)	Suburb (Mean ± SD)	Town (Mean ± SD)	Rural (Mean ± SD)	*p*-Value	Partial Eta Squared
Calories	620 ± 18	624 ±18	615 ± 23	610 ± 23	0.473	0.032
Protein (g)	30.5 ± 0.4	30.0 ± 1.4	30.4 ± 0.7	30.5 ± 0.8	0.855	0.010
Carbohydrate (g)	76.4 ± 1.6	75.8 ± 4.3	74.5 ± 3.2	74.8 ± 4.1	0.584	0.025
Total Fiber (g)	8.0 ± 1.0	7.2 ± 0.4	7.7 ± 0.5	7.6 ± 0.5	0.539	0.028
Sugar (g)	33.8 ± 1.6	33.9 ± 2.0	31.4 ± 1.8	31.8 ± 2.2	0.025	0.113
Added Sugar (g)	0.6 ± 0.7	1.6 ± 1.6	0.2 ± 0.4	0.2 ± 0.4	0.002	0.171
Total Fat (g)	22.7 ± 1.6	23.3 ± 1.4	22.6 ± 1.6	21.9 ± 1.2	0.114	0.074
Saturated Fat (g)	8.0 ± 0.3	8.1 ± 0.3	8.3 ± 0.7	8.1 ± 0.5	0.311	0.045
MUFA (g)	5.5 ± 1.2	6.2 ± 0.3	5.7 ± 0.6	5.6 ± 0.5	0.405	0.037
PUFA (g)	3.7 ± 1.0	4.0 ± 0.1	3.5 ± 0.5	3.3 ± 0.4	0.081	0.083
Trans Fat (g)	0.5 ± 0.2	0.6 ± 0.1	0.6 ± 0.2	0.6 ± 0.1	0.768	0.015
Cholesterol (mg)	61.9 ± 5.4	66.5 ± 3.1	69.0 ± 7.5	66.7 ± 5.3	0.101	0.077
Vitamin A (IU)	3838.1 ± 1270.4	3149.3 ± 1525.3	3403.5 ± 1090.0	3366.3 ± 1004.6	0.785	0.014
Thiamin (mg)	0.37 ± 0.04	0.40 ± 0.01	0.37 ± 0.03	0.38 ± 0.03	0.486	0.031
Riboflavin (mg)	0.74 ± 0.03	0.77 ± 0.02	0.73 ± 0.04	0.74 ± 0.03	0.511	0.029
Niacin (mg)	5.32 ± 1.10	5.48 ± 0.49	5.49 ± 0.66	5.45 ± 0.53	0.917	0.007
Vitamin B6 (mg)	0.52 ± 0.06	0.49 ± 0.02	0.51 ± 0.05	0.50 ± 0.04	0.731	0.017
Vitamin B12 (mcg)	1.77 ± 0.15	1.90 ± 0.12	1.86 ± 0.13	1.89 ± 0.10	0.224	0.055
Biotin (mcg)	1.78 ± 0.70	1.91 ± 1.33	1.76 ± 0.69	1.80 ± 0.71	0.746	0.016
Pantothenic Acid (mg)	1.84 ± 0.18	1.82 ± 0.12	1.78 ± 0.10	1.79 ± 0.10	0.560	0.026
Folate (mcg)	85.34 ± 15.20	87.53 ± 5.44	75.44 ± 8.11	75.96 ± 7.55	0.046	0.098
Vitamin C (mg)	29.69 ± 7.26	27.58 ± 2.00	26.48 ± 7.21	27.44 ± 5.93	0.657	0.021
Vitamin D (IU)	10.33 ± 3.09	10.52 ± 5.45	9.29 ± 3.78	8.72 ± 3.65	0.378	0.039
Vitamin E (mg)	1.61 ± 0.43	1.61 ± 0.28	1.49 ± 0.24	1.52 ± 0.19	0.582	0.025
Vitamin K (mcg)	37.53 ± 12.90	31.82 ± 9.09	26.84 ± 7.45	30.46 ± 7.82	0.036	0.104
Calcium (mg)	517.7 ± 47.6	497.5 ± 23.6	497.5 ± 17.3	495.7 ± 23.7	0.057	0.092
Fluoride (mg)	0.02 ± 0.00	0.02 ± 0.00	0.02 ± 0.00	0.02 ± 0.00	0.355	0.041
Iron (mg)	3.44 ± 0.19	3.59 ± 0.17	3.57 ± 0.20	3.58 ± 0.25	0.511	0.029
Magnesium (mg)	97.00 ± 7.00	88.82 ± 6.74	92.23 ± 4.74	92.79 ± 5.17	0.367	0.040
Phosphorus (mg)	532.5 ± 34.0	519.8 ± 10.2	514.7 ± 22.3	516.7 ± 24.8	0.450	0.034
Potassium (mg)	1024.6 ± 46.8	990.3 ± 43.6	1019.4 ± 43.7	1023.5 ± 57.7	0.929	0.006
Sodium (mg)	1125.6 ± 119.1	1082.6 ± 80.6	1089.9 ± 89.8	1090.8 ± 85.3	0.536	0.028
Zinc (mg)	3.64 ± 0.30	3.48 ± 0.36	3.63 ± 0.25	3.77 ± 0.29	0.153	0.066
HEI ^^^	61.5 ± 2.6	61.5 ± 1.3	62.6 ± 3.5	61.8 ± 2.3	0.571	0.026

^^^ HEI score out of 100 points maximum, with a higher score indicating higher DQ.

## Supplementary Materials

The following are available online at https://www.mdpi.com/1660-4601/17/21/8012/s1, Supplement S1: Menu portioning assumptions, Table S1: ESHA codes used for nutrient analysis, Supplement S2: HEI calculator instructions and equations for DQ analysis.
