# Supplementary material for: Evaluation of Variability in Dietary Quality of School Lunches Meeting National School Lunch Program Guidelines by Socioeconomic Status and Rurality
Source: Int J Environ Res Public Health. 2020 Oct 30;17(21):8012. doi: 10.3390/ijerph17218012 (PMC7663583; doi:10.3390/ijerph17218012)
Supplement: Supplementary file 1 [file ijerph-17-08012-s001.pdf]

International Journal of *Environmental Research and Public Health*

MDPI

Supplement 1. Menu portioning assumptions

1. Start with the first full week. If there is a week with missing days, use the non-full week's days to fill in the missing days.
2. If main dish is a combination of meat/meat alternate and grain components, provide enough to ensure that 2oz meat/meat alternate or 14g protein and 2oz grain or 30g carbohydrate.
3. If multiple options for the fruit, based on meal planning principles of variety and flavor pairing, choose what complements the meal flavor or week variety best.
4. If multiple options for the vegetable, choose what needs to be met still for vegetable variety NSLP requirements.
5. If multiple entrees/ lunches, decide if you will use the first, second, etc. and consistently use that ordered entree/ lunch.
6. Salad = 1c salad, 1 T dressing, choose appropriate dressing to compliment flavor of meal if no dressing specified
7. Steamed vegetables = add 1t butter
8. If something has cheese sauce, gravy, or dip, give 1/8c or 2 T.
9. Roll not as a sandwich bun = add 1 t butter
10. Peanut butter as 2oz meat alternate = 4 T
11. Yogurt as 2oz meat alternate = 1c
12. Burrito = 1 1/2oz tortilla, 1/2oz rice, 2oz ground beef if meat/ meat alternate not specified
    a. If smothered, add 1/4c salsa, 1 T reduced-fat sour cream, 1 T cheese sauce
13. Tacos = assume hard unless otherwise specified, assume ground beef if meat/ meat alternate not specified, add 1/8t taco seasoning
14. Stir-fry = 2oz meat, 3/4c Asian medley vegetable, 1t oil, 1t soy sauce, 1t teriyaki sauce
15. Super nachos = 2oz ground beef, 2T cheese sauce, 2oz chips
16. Roasted vegetables = squash if not specified, choose squash type to meet vegetable variety required for week, add 1t oil
17. Taco salad = 1c lettuce, 1/4c salsa, 1oz cheddar cheese, 1oz ground beef, 1/8t taco seasoning
18. Sancho = burrito + 1/4c enchilada sauce

19. Taco burger = 1 1/2oz ground beef, 1/2oz cheddar cheese, 1/8t taco seasoning
20. Tater tot casserole = 1oz egg, 1oz cheddar cheese, 3/4c tater tots
21. Cowboy cavatini = 2oz penne, 1/4c marinara sauce, 1 1/2oz ground beef, 1/2oz mozzarella cheese, 1/8t taco seasoning
22. Enchilada = assume beef if not specified
23. Fajita = 2oz meat, 2oz tortilla, 1/4c onion, 1/4c green pepper, 1/4c red pepper, 1/4t fajita seasoning, 1t oil
24. Fajita or burrito bowl = swap out tortilla and use 2oz rice for grain
25. Strawberries and bananas for fruit = 1/4c fresh strawberries + 1/4c fresh banana
26. Grilled cheese = 2oz bread, 2oz American cheese, 2t butter
27. Cheeseburger = 1 1/2oz ground beef, 1/2oz cheese
28. Pork carnitas = soft taco shell for grain, pulled pork for meat
29. Mac and cheese with an additional protein source (fish sticks, meatballs, little smokies) = provide at least 1oz of additional protein source
30. Apple crisp for fruit = applesauce
31. Spaghetti pie = 2oz mozzarella cheese, 2oz spaghetti noodles, 1/4c marinara
32. Pancake = add 1T syrup
33. Pigs in a blanket = 2oz hot dog, 2oz crescent roll
34. Spaghetti = use 1/4c marinara sauce
35. Walking tacos = 2oz Fritos, 1 1/2oz ground beef, 1/8t taco seasoning, 1/2oz cheddar cheese, 1/4c lettuce, 1/4c salsa
36. Meat sauce = amount of ground beef needed for meal + 1/4c marinara sauce
37. Beef and noodles = 2oz ground beef, 2oz egg noodles, 1/4c beef gravy
38. Chicken and noodles = 2oz grilled chicken, 2oz egg noodles, 1/4c chicken gravy
39. Cheesy bread stick = provide 1/4c marinara for dipping
40. Beef wrap = 2oz roast beef lunch meat, 2oz tortilla
41. Chili = assume beef
42. Pasta bake = 2oz rigatoni, 1/4c marinara sauce, 2oz mozzarella cheese
43. Italian hot ham and cheese = 2oz Italian bread, 1oz ham, 1oz provolone, 1 T Italian dressing

44. Frito pie = 2oz Fritos, 1/2oz cheddar cheese, 10g protein from chili
45. Apple salad = 1/2c apple, 2 T vanilla NF yogurt
46. BBQ chicken = 2oz grilled chicken + 2T BBQ sauce
47. Meatball sub = 2oz meatballs, 2oz bun, 2T marinara sauce
48. Beef taco supreme = 2oz hard taco shell, 1 1/2oz ground beef, 1/8t taco seasoning, 1/2ox ground beef
49. Sloppy Joe = 2oz ground beef, 1/4c sloppy Joe sauce
50. Chicken Alfredo = 2oz linguini, 2oz grilled chicken, 1/4c Alfredo sauce
51. Cowboy beans = use baked beans
52. Sloppy nachos = 2oz ground beef, 2T sloppy Joe sauce, 2T cheese sauce, 2oz tortilla chips
53. Taco crunch = beef hard taco
54. Sausage + gravy = 2oz sausage + 2T white gravy
55. Cowboy cornbread = chili + cornbread
56. Smothered steak = 2oz steak, 2T mushroom gravy
57. Tater tot enchilada bake = 1 1/2oz ground beef, 1/2oz cheddar cheese, 2T enchilada sauce, 3/4c tater tots
58. Chicken and waffles = chicken tenders in amount to reach 14g protein, at least 1 1/2oz waffle, 2T syrup
59. Fish taco = 2oz soft tortilla, 2oz tilapia, 1/4c coleslaw
60. Pizza quesadilla = 2oz tortilla, 1 1/2oz mozzarella cheese, 1/2oz pepperoni
61. Chef salad = 2c lettuce, 1/2 egg, 1/2oz cheddar cheese, 1/2oz ham, 1/2oz turkey, 2T Italian dressing
62. Sidekick for fruit = use juice
63. French bread pizza = 2oz French bread, 2oz mozzarella cheese, 1/4c marinara sauce
64. Gran's fruit salad = 1/8c each banana, grapes, strawberries, mandarin oranges + 2T vanilla pudding
65. Tri-tater = use tater tots
66. Chili dog = 2oz hot dog, 1/4c chili
67. Cheesesteak = 1oz sirloin, 1oz provolone
68. Roasted vegetable/ potatoes = add 1t oil

Roasted vegetable = use whatever vegetable needed to meet vegetable variety requirement for the week (butternut squash, yellow squash, zucchini)

Table 2. ESHA codes used for nutrient analysis.

Vegetable	Code	Fruit	Code	Grain	Code
Salad, garden	78311	Grapes, fresh	71089	roll, white	71351
Broccoli, fresh	5556	Apple, fresh	3002	roll, whole grain	42057
Broccoli, frozen	5030	Banana, fresh	3021	biscuit, plain	71182
Cauliflower, fresh	5050	Orange, fresh	3083	biscuit, whole grain	78962
Cauliflower, frozen	5053	Apple, canned use applesauce	16419	cornbread/muffin	42116
California mix veg, frozen	63991	Applesauce	16419	breadstick	71259
Green beans, fresh	5009	Pineapple, canned	71114	garlic toast	72857
Green beans, frozen	5013	Mandarin Orange	71773	blueberry muffin	15480
Celery, fresh	5054	Jello Fruit cup (1 individual cup	78367	cinnamon roll	38912
Carrots, fresh	15304	Strawberries, fresh	3135	oatmeal raisin cookie	47003
Carrots, frozen	5358	Peaches, canned	71051	chocolate chip cookie	47001
Peas & Carrots, frozen	5123	Pears, canned	9897	pita bread, white	71227
Pea	5118	Tropical fruit, canned (1 individ	71961	pita bread, whole grain	71228
Corn	16944	Fruit cocktail	3164	english muffin, whole grain	93724
Spinach, salad	78410	Strawberries, frozen	3137	english muffin, white	42289
Spinach, cooked (add oil/garlic)	5148	Cantaloupe, fresh	3075	pancakes, white	45066
Pepper, green, fresh	6844	Honeydew, fresh	3080	pancakes, whole grain	45008
Pepper, red, fresh	5295	Blueberries, fresh	3029	french toast sticks, white	42354
Cucumber	5071	Blueberries, frozen	3031	waffles, white	45209
Squash, roasted veg.	5317	Rasberries, fresh	3648	waffles, whole grain	67292
Asian veg (no additions)	66157	Rasberries, frozen	78714	french toast sticks, whole grain	79055
Black Beans (7g pro = 4.5oz.)	9262	Blackberries, fresh	3924	granola (1.5 oz)	40063
Baked Beans	7038	Blackberries, frozen	3028	tortilla chips	17395
Refried beans	17370	Watermelon	3142	fritos	44278
Pinto beans	7051	Kiwi	27502	hard taco shell	42443
Lettuce, plain	5083	Apple, salad	95944	soft taco shell, white	33427
Tomato, cherry	15327	Juice cup, frozen (4oz OJ)	78377	soft taco shell, whole grain	33484
Sweet potato, baked	5542	Raisins	3130	tortilla, white	33427
Sweet potato, puff	67301	Craisins	3487	tortilla, whole grain	33484
Sweet potato, tater tot	67301	Summer fruit salad (.25 side)	29647	hot dog bun, white	42021
Sweet potato, fries	41749	Plum, fresh	3123	hot dog bun, whole grain	93716
Potato, mashed	93812	Apricot halves, canned	3152	hamburger bun, white	42020
Potato, baked (3 oz.)	5338	Apple crisp (.75c)	45532	hamburger bun, whole grain	93715
Potato, French fries (3oz)	41742	Pears, fresh	3104	french bread, white	55363
Potato, tater tots (3oz)	17852	Peach, fresh	3097	sandwich bread, white	71242
Potato, hasbrown (3oz)	17852	Pineapple, fresh	3111	sandwich bread, whole grain	93801
Potato, tri tator (3oz)	17852	Apple, baked use applesauce	16419	pretzel bun	42093
Potato, au gratin	83204	Apricots, fresh	3657	rice, white (.5c)	38013
Potato, scalloped	5270	mango, fresh	3220	rice, brown (.5c)	38010
Potato salad	56005	Avocado	3016	savory rice (.5c)	82874
Cole Slaw	5461	banana cream pie	88022	spaghetti noodles, white	38118
Zucchini	5598	Apple,juice,unsweetened	3008	spaghetti noodles, whole grain	93626
Onion	5102	Nectarine	3216	linguini, white	38118
Snow peas	13960	cherries	3036	linguini, whole grain	93626
Tomato Soup (1 cup)	50028	Italian Ice (0.5 c)	25891	penne, white	94103
Glazed carrots	5633	Orange juice	21113	penne, whole grain	93623
breaded fried broccoli	5513			rotini, white	94068
Beets	5022			rotini, whole grain	93624
cauliflower poppers (124g)	5539			rigatoni, white	82854
Yellow Squash	82848			egg noodles, white	38047
breaded fried broccoli	5513			goldfish	93767
Lentils	7006			saltine crackers	43506
Black eyed peas (frozen)	4438			mexican rice	78701
Roasted Potato + (add 1 tsp oil)	5338			fried rice	23938
Kale salad	5208			Crescent roll(2.75 oz=2 oz)	16638
Broccoli Slaw + (add 2T 44704)	78317			apple cake	46098
Curly Fries (3 oz)	8987			italian bread	71219
Edamame	9929			spanish rice	78725
Romaine salad	5088			cinnamon chips	14174
Butternut squash	5317			stuffing (6oz)	42037
Lima beans	5019			egg noodles, WG (1.5oz)	59781
Broccoli salad	95947			Birthday cake (0.85 pieces)	46015
				lo mein	83362
				cheese biscuit (4 oz)	24646
				Flatbread (WG)	67267
				Cavatappi (1.5 oz)	59738
				whole wheat soft pretzel	24400
				Pretzel	44015
				Lemon Poppy Seed Muffin (1 each)	83241
				Cheez-It	43661
				oat roll	42070
				whole grain crackers	43508
				oatmeal raisin cookie	47003

Protein	Code	Extras	Code	Combo foods	Code
Lit'l smokies	13232	Ranch	44696	Macaroni and cheese (6oz)	33948
Cheese, cheddar	47863	Italian	8020	Lasagna (1 cup)	83202
Hamburger	58125	Butter	8000	Meatloaf (2 oz.)	94216
Chicken Nuggets (4oz)	38954	Oil (1t)	44975	Bierock	2oz roll 71351,
Pork Cutlet (breaded)	12087	Garlic (.25t)	9473	Corndog (2 each)	38953
Chicken popper/popcorn (3 oz.)	76484	Terykari sauce	9531	Pizza, cheese (0.9slice)	93674
Chicken giggles	38954	Sweet and sour red sauce-	26459	Pizza, pepperoni (0.9 slice)	93676
Chicken tenders/fingers (3.5oz)	52664	Marinara	39134	Stromboli (0.5each)	82711
Chicken patty, breaded (3.5oz)	14728	Salsa	91049	Buffalo chicken calzini	
Chicken, grilled	67033	Cheese sauce	53523	Chicken cacciatore (0.5 entrée)	82920
Chicken drumstick, breaded (1.25 each)	76339	Gravy, beef	53023	Goulash	1oz ground bee
Pork riblet	68905	Gravy, chicken	53022	Chicken tetrazzini (1.1c)	56199
Pork loin	12063	Fajita seasoning (.25t)	91932	Cheese breadstick (4oz) (2 Each)	78770
Pork chop, breaded	12081	Taco seasoning (.125t)	66958	Bosco stick (2 each)	78770
Pork, pulled	76508	Jelly	23294	Crispitos (1.5)	77061
Chicken salad	82752	Syrup	25002	Cheesy pull-aparts	
Tuna salad	82753	Alferdo sauce	82679	Beef Stroganoff (1.1entree)	82816
Egg salad	52066	Gravy, sausage (1/4c)	92571	Enchilada, chicken (2each)	83331
Hummus	7957	Guacamole	20771	Chicken parmesan (no noodles,	78715
Peanut butter	4627	Sour cream	54380	Cheeseburger hot pocket (1.25)	57776
Yogurt	89921	BBQ sauce	53000	Beef & noodles (.5pkg)	16400
String cheese	48252	White gravy	68546	Beef tamale (pie) 1.4 each	16811
Fish patty, breaded (4oz.)	67356	Honey	25309	Ravioli (2c, no extra marinara needed)	38956
Fish sticks, breaded	17002	Enchilada Sauce	4498	Sausage pizza (0.85 piece)	93678
Fish, grilled/baked	52517	Chili Seasoning	66959	Rock and Roll Beef Wrap	2 oz tortilla 334
Cheese, Mozz.	47889	Caesar dressing	44705	Egg roll, chicken (4.75 oz.)	15271
Cheese, American	1000	Poppy Seed dressing	38839	pizza, cheese, stuffed crust (0.6 slice)	28397
Cheese, swiss	1071	queso cheese (30 g)	51842	cheese tortellini	92216
Cheese, provalone	47899	Cajun seasoning	91947	beef enchilada (2 each)	88791
meatballs (3.5 oz)	78688	Vanilla Pudding	2657	Mexican pizza (0.5 whole pizza)	17683
Ham, baked	12314			Taco soup (1 cup + 1oz. Beef + 1oz. Cheese	40692
Turkey luncheon meat	15736			Pepperoni Calzone (0.67 each)	18638
Ham luncheon meat	13263			Ham and Cheese Hotpocket (1.25 each)	70920
Roast beef luncheon meat	89758			Sancho	1.5 oz tortilla 3
Sausage	13269	**Dairy**	**Code**	pizza, pepperoni, stuffed crust (.5 individua	89306
Hot dog	48642	1% milk	18760	Chili	28167
Ground beef	58125			Pepperoni Hot Pocket (1 each)	18625
Sirloin steak	39074			Fiesta Chicken Pasta	23777
Salsibury steak (7.5oz), no extra gravy	93824			Fried Mozarella sticks (4 each)	23964
Country fried steak (.75 each)	57943			Chicken tortilla soup (1 cup + 1.5oz. chicke	40692
Steak/beef fingers (2.5oz)	11708			Tuna noodle casserole (6 oz.)	83335
Bacon	51151			Uncrustable WG	80031
Eggs, scrambled (4oz=14g prot)	19516			Hamburger casserole	6oz mac and ch
Chicken, teriyaki	15915			Breakfast pizza (1.5 slice)	83373
Chicken, sweet and sour (4 oz)	66875			Egg roll, pork (1.75 each)	14971
Chicken, general tso (5oz)	83366			Cheeseburger pizza (1 slice)	78896
Sloppy joes sauce (1/8c) add grn beef	53714			Chicken bacon ranch pizza (1.25 slice)	48735
Turkey, baked	16038			BBQ chicken pizza (.75 slice)	82893
Chicken Parmesan	78715			Chicken taquito (3 each)	67308
Chili, beef (3/4c)	28167			Chicken cordon bleu (3 each)	76948
Chicken nuggets, whole grain	76501			Pizza poppers/ crunchers (8 each)	91615
Chicken, orange (4oz.)	83319				
Shrimp poppers (4oz)	23956				
Chicken fried steak (3oz)	57943				
Pork fritter	12081				
White chicken chili (1 indv. Cup)	33280				
Cowboy cavatini					
BBQ beef (4 oz)	58383				
BBQ chicken leg (4 oz)	68490				
Breaded beef patty (3oz)	11623				
Baked chicken leg	15154				
Roast beef	10281				
Pepperoni (7g pro = 1.25 oz.)	13021				
Salami (7g pro = 2 oz.)	13023				
cheese, parmesan	1075				
Turkey burger	26795				
Hash, beef (4.5 oz.)	56150				
Ham patty	12169				
wings, boneless, spicy	76469				
Deviled Egg (2 each)	24640				
Bratwurst	58010				
Pork chop	38899				
Spicy chicken	77163				

Supplement 2. HEI calculator instructions and equations for DQ analysis

<u>Instructions:</u>

1. Sum all nutrients for each day to obtain a daily total for each nutrient.

2. Copy and paste HEI equations at the end of those sums.
3. Fill in the amounts at beginning of calculator – whole fruit (c), dark green veg (c), whole grain (oz.), seafood/ plant protein (oz.).
   a. If menu does not say whole grain, assumed products were white or whole grain-rich and received 0 for whole grain section (except for corn grain products).
4. Score amounts at end of calculator using scoring scale below for fatty acid ratio, sodium, and saturated fat.

FA Ratio	FA Score	Sodium	Na Score	Sat Fat	SF Score
2.5	10	1.1	10	8	10.0
2.4	9.1	1.2	9	8.5	9.0
2.3	8.4	1.3	8	9	8.4
2.2	7.7	1.4	7	9.5	7.8
2.1	7.0	1.5	6	10	7.2
2.0	6.3	1.6	5	10.5	6.6
1.9	5.6	1.7	4	11	6.0
1.8	4.9	1.8	3	11.5	5.4
1.7	4.2	1.9	2	12	4.8
1.6	3.5	2.0	1	12.5	4.2
1.5	2.8			13	3.6
1.4	2.1			13.5	3.0
1.3	1.4			14	2.4
1.2	0.7			14.5	1.8
				15	1.2
				15.5	0.6
				16	0.0

5. Check that no scores at end of calculator (cell columns CN-CZ) are over the max HEI score for that component.

   a. Max scores = total fruit 5, whole fruit 5, total vegetable 5, dark green/ legume 5, whole grain 10, dairy 10, total protein foods 5, seafood/ plant proteins 5, fatty acid ratio 10, refined grain 10, sodium 10, added sugar 10, saturated fat 10

6. Check that HEI score (cell column BM) is not over 100.

<u>Excel Calculator Equations:</u>

*The Excel calculator requires three sets of cells to transform input data from nutrient analysis and menu into the HEI score. The three cells are consecutively linked and build off of each other.

General Cell Rationale:

1. First cell = amount of that nutrient of food group in the lunch
   a. Some first cells were automatically completed for all lunches due to the NSLP nutrition standards. Every lunch must contain 0.5c fruit, 0.75c vegetable, 1c dairy, 2oz protein, 0 refined grain, and minimal added sugar (full credit given to all lunches).
2. Second cell = (first cell)*1000/(cell with calorie data from nutrient analysis)
   a. The HEI score is per 1000 calories, so the first cell must be standardized to 1000 calories using a ratio.
3. Third cell = (second cell)/(amount to receive max score per HEI-2015)*(max score for the component)
   a. This is the actual score the lunch received for this HEI scoring component. Because we were unable to put a maximums or minimums on this equation, researchers needed to check all third cells to ensure that they did not exceed that HEI scoring components' max score (instructions #4 and #5 above).
